# An Analysis of Physiology; Being a Condensed View of Its More Important Facts and Doctrines

**Published:** 1847-10

**Authors:** 


### ARTICLE XI.

*An Analysis of Physiology; being a condensed view of its more important facts and doctrines.* Designed especially for the use of Students. By John J. Reese, M. D., Lecturer on Materia Medica in the Medical Institute of Philadelphia; Fellow of the College of Physicians; Secretary of the Philadelphia Medical Society. Philadelphia; J. G. Auner. 1847. pp. 314, 12 mo. (From the Author. For sale by Joseph Keen, Jr. Chicago.)

We have, several times, expressed our doubts of the expediency of furnishing to students of medicine, works of an exceedingly condensed character, and *intended to facilitate* their studies. That it has the *tendency* to make them superficial, by removing the necessity for the study of larger and more explanatory works, but few will doubt. The form of Catechism is, however, much more objectionable than that of simple condensation. The work before us being of the latter description is, on that account, less objectionable than it otherwise would be. We should also fairly state, that for its size it is very comprehensive, and would, if used *only* as a means of review by students attending a course of Lectures on Physiology, doubtless be a sufficient introduction to the study. For such purpose we would recommend it as affording a good synopisis of this department of medicine. The most recent works have been used by the author in preparing his little work, and to him the credit is due of having well performed the rather difficult task of condensation, without loss of perspicuity. Dr. Reese has had some experience in the conduct of examinations and instruction of students, and is consequently well qualified to judge of the points most essential to retain to preserve a correct and complete outline of the science.

J. V. Z. B.

